# ACK1 Contributes to the Pathogenesis of Inflammation and Autoimmunity by Promoting the Activation of TLR Signaling Pathways

**DOI:** 10.3389/fimmu.2022.864995

**Published:** 2022-05-20

**Authors:** Lina Jing, Xin Zhang, Dong Liu, Yonghong Yang, Huabao Xiong, Guanjun Dong

**Affiliations:** ^1^Cheeloo College of Medicine, Shandong University, Jinan, China; ^2^Institute of Immunology and Molecular Medicine, Jining Medical University, Jining, China; ^3^School of Medical Laboratory, Weifang Medical University, Weifang, China; ^4^Department of Clinical Laboratory, Affiliated Hospital of Jining Medical University, Jining, China; ^5^Medical Research Center, Affiliated Hospital of Jining Medical University, Jining, China; ^6^Jining Key Laboratory of Immunology, Jining Medical University, Jining, China

**Keywords:** ACK1, TLR, macrophages, dendritic cells, inflammation, autoimmunity

## Abstract

Toll-like receptors (TLRs) are the first line of defense in the immune system, whose activation plays a key role in the pathogenesis of inflammation and autoimmunity. TLRs can activate a variety of immune cells such as macrophages and dendritic cells, which produce proinflammatory cytokines, chemokines, and co-stimulatory molecules that lead to the development of inflammation and autoimmune diseases. As a nonreceptor tyrosine kinase, ACK1 is involved in multiple signaling pathways and physiological processes. However, the roles of ACK1 in the activation of TLR pathways and in the pathogenesis of inflammation and autoimmune diseases have not yet been reported. We found that the expression of ACK1 could be upregulated by TLR pathways *in vivo* and *in vitro*. Intriguingly, overexpression of ACK1 significantly promoted the activation of TLR4, TLR7, and TLR9 pathways, while knockdown of ACK1 or the use of the ACK1 inhibitor AIM-100 significantly inhibited the activation of TLR4, TLR7, and TLR9 pathways. *In vivo* studies showed that the inhibition of ACK1 activity by AIM-100 could significantly protect mice from the TLR4 agonist lipopolysaccharide (LPS)-mediated endotoxin shock and alleviate the condition of imiquimod-mediated lupus-prone mice and MRL/*lpr* mice. In summary, ACK1 participates in TLR-mediated inflammation and autoimmunity and has great potential in controlling inflammation and alleviating autoimmune diseases.

## Introduction

Toll-like receptors (TLRs) are integral membrane-bound receptors with a unique characteristic of possessing the highly conserved pattern recognition receptor (PRR) (1–3). TLRs are important as they can recognize distinct patterns of antigens and act as the first line of defense in innate immunity (4, 5). TLRs bind to corresponding ligands within the myeloid cells that eventually leads to inflammation, and play an indispensable role in the modulation of autoimmune diseases (6, 7). Hence, TLRs are a type of immune receptor whose activation is linked to the mediation of inflammation and autoimmunity, and they play an important role in controlling inflammation and autoimmunity (8–10).

Macrophages and dendritic cells (DCs) have an irreplaceable role in the immune system (11). Specifically, macrophages mediate phagocytosis and antigen presentation, and the main functions of DCs are antigen presentation and cytokine production (11, 12). TLRs are the key components of innate and adaptive immune responses and are mainly expressed on antigen-presenting cells such as macrophages and DCs (13–16). TLRs increase the production of proinflammatory factors and reduce the production of anti-inflammatory factors through specific factors in macrophages (17). TLRs function by activating macrophages and DCs to act as a “goalkeeper” of the immune system in humans and animals, thereby protecting the host from autoimmune diseases and inflammation (18). Inappropriate stimulation of TLRs may activate the signaling pathways of nuclear factor kappa-light-chain-enhancer (NF-kB) and mitogen-activated protein kinase (MAPK), resulting in strong systemic inflammation and the recruitment of a large number of immune cells in the body (19, 20). According to previous studies, by combining with a suitable ligand, TLRs can participate in the regulation of autoimmunity by macrophages and DCs through the primary response of the myeloid cell differentiation 88 (MyD88)-dependent pathway and other regulatory protein pathways (20, 21). Therefore, it is very important to elucidate the pathological mechanisms of autoimmune diseases and inflammation related to TLR signaling pathways.

Activated CDC42-associated kinase 1 (ACK1) is a nonreceptor tyrosine kinase encoded by the *TNK2* gene in humans (22–24). It has been confirmed that ACK1 is associated with immune cell endocytosis, survival, proliferation, and migration by combining with a variety of receptor tyrosine kinases (25, 26). ACK1 plays an important physiological function in the immune system (21, 27). Overexpression of ACK1 activates Akt through proinflammatory factors released by immune cells, which could promote cell proliferation and apoptosis deregulation, thereby transforming chronic inflammation into cancer (26). Previous studies have indicated that ACK1 as a kinase binds to the corresponding domains of certain phosphorylated proteins on immune cells and regulates their phosphorylation (28, 29). Although ACK1 is involved in multiple signaling pathways and physiological processes, the regulatory effect of ACK1 on the activation of the TLR signaling pathway and its mediating role in inflammation and autoimmunity have not yet been reported.

Considering that dysfunction or dysregulation of TLRs, especially TLR4, TLR7, and TLR9, leads to a series of pathological conditions (29–31), we investigated whether ACK1 regulates the activation of the TLR signaling pathway and participates in the pathogenesis of inflammation and autoimmunity. In the present study, we found that ACK1 promoted the activation of TLR4, TLR7, and TLR9 pathways in macrophages and DCs. Notably, pharmacological inhibition of the activity of ACK1 not only alleviated the conditions of TLR4-mediated endotoxic shock mice but also relieved the conditions of TLR7-mediated lupus model mice. Taken together, our study demonstrates that ACK1 participates in the regulation of TLR-mediated inflammation and autoimmunity.

## Methods and Materials

### Mice

Wild type C57BL/6 mice (female; 6–8weeks; purchased from Pengyue Experimental Animal Breeding Co. Ltd) were housed under specific pathogen-free conditions at Jining Medical University throughout the experimental period. Female, MRL/*lpr* lupus-prone mice were purchased from Nanjing University Model Animal Research Centre. MRL/*lpr* mice received weekly intraperitoneal injections with 20 µg/g of AIM-100 (Selleck) from the 14^th^ week to the 18^th^ week. Animal care and experiments were conducted in accordance with the institutional guidelines.

### Antibodies

Anti-mouse β-actin (AF0003; 1:1,000 dilution), Horseradish peroxidase (HRP)-anti-rabbit immunoglobulin G (IgG) (A0208; 1:3,000 dilution) and HRP-anti-mouse IgG (A0216; 1:3,000 dilution) were purchased from Beyotime Institute of Biotechnology (Haimen, China). Anti-ACK1 (PA5-102625) antibody was purchased from Invitrogen (Carlsbad, CA, USA). Anti-p-p65 (9246), anti-p65 (9242), anti-p-Erk (9101), anti-Erk (9107), anti-p-JNK (9251), anti-JNK (9251), anti-p-p38 (4511) and anti-p38 (9228) antibodies were purchased from Cell Signaling Technology (Danvers, MA, USA). For flow cytometry purposes, fluorochrome-conjugated anti-B220 (Cat#: 103206), anti-CD4 (Cat#: 100406), anti-CD40 (Cat#: 124610), anti-F4/80 (Cat#: 123108), anti-CD86 (Cat#: 105012), anti-CD11c (Cat#: 117310), anti-GL7 (Cat#: 144608), anti-CD95 (Cat#: 152604), anti-CXCR5 (Cat#:145506), and anti-PD-1 (Cat#:135206) antibodies were purchased from BioLegend. Unless specifically listed otherwise, all flow cytometry antibodies were stained at a 1:100 dilution.

### Bone Marrow-Derived Macrophages (BMDMs) and Bone Marrow Derived Dendritic Cells (BMDCs)

Briefly bone marrow cells were isolated from tibias and femurs of 6-weeks-old C57BL/6 mice. BMDMs were grown in complete Dulbecco’s Modified Eagle’s Medium (DMEM, Gibco) and BMDCs were grown in complete Roswell Park Memorial Institute (RPMI) 1640 Medium (Gibco) for 7 days. Where indicated, the BMDMs were stimulated with recombinant mouse granulocyte macrophage colony stimulating factor (GM-CSF, 10 ng/mL, Peprotech) for BMDMs differentiation, the BMDCs were stimulated with GM-CSF (10 ng/mL) and recombinant mouse lnterleukin-4 (1 ng/mL, Peprotech). All cell culture was done at 37°C with 5% CO_2_. LPS stimulation was performed with lipopolysaccharide (LPS, L6511; Sigma-Aldrich) for the indicated time. As ACK1 inhibitor, AIM-100 (T3434; Sigma-Aldrich) were added to cells for 2 hours before LPS treatment. Cells were harvested at a density of 2 × 10^6^ cells/ml for experiments.

### Cell Viability Assay

Cell viability was monitored with CCK8 kit (CCK8, Dojindo, Japan) following the producers suggestions. A total of 1×10^5^ BMDMs and BMDCs were seeded in 96-well plates and treated with various doses of AIM-100 for 48 hours. After incubation, CCK8 solution (10 μL) were added to each well and incubated at 37°C for 1 hour. Subsequently, the absorbance was measured at 450 nm using a microplate reader (HTX; Biotek, Beijing, China).

### Murine Model of Endotoxic Shock

Endotoxemia was induced by intraperitoneal injection into the mice of LPS. To set up a mouse model of lethal endotoxic shock, C57BL/6 female mice aged 8 weeks were pretreated with an i.p. injection of 5, 10, and 20 µg/g of body weight of AIM-100 or vehicle (saline) administered 2 hours before an i.p. injection of 37.5 µg/g of body weight of LPS. The survival of individual mice was monitored up to 3 days. In the low-dose LPS model, mice were pretreated with an i.p. injection of 5, 10, and 20 µg/g of body weight of AIM-100 or vehicle (saline) administered 2 hours before an i.p. injection of 10 µg/g of body weight of LPS. Blood was collected after 3 hours, non-heparinized 1.5 mL Eppendorf tube and livers, lungs, and spleens were collected after 12 hours. Blood was processed and serum aliquots were stored at −20°C for cytokine analysis.

### Generation of Imiquimod-Induced Lupus Model

Female C57BL/6 mice (8-week-old) were treated topically with the TLR7 agonist imiquimod, which induces systemic autoimmunity with lupus-like characteristics in various non–autoimmune-prone mice strains. A 5% imiquimod cream (Med-Shine Pharmaceutical, China) was applied topically to the right inner ear three times a week for 10 weeks. During the 10 week of treatment, IMQ-induced lupus-prone mice received weekly intraperitoneal injections with 20 µg/g of AIM-100. The mice were killed and disease severity was analyzed after 10 weeks from the onset of treatment.

### Quantitative Real-Time PCR Analysis

The total RNA was isolated using RNAiso Plus reagent (TaKaRa) and reverse transcribed into cDNA using reverse transcription kit (Vazyme Biotech). Quantitative real‐time PCR analysis was performed using real‐time PCR kit (Vazyme Biotech). The mixture was prepared by mixing 12.5 µL of Maxima SYBR Green qPCR Master Mix, 0.3 µM of forward and reverse primers and ≤ 500 ng of cDNA. Nuclease-free water was used to top up the mixture up to 25 µL. The reactions were incubated in a 96-well plate at 95°C for 10 min followed by 40 cycles of 95°C for 15 s, 60°C for 30 s, and 72°C for 30 s and then 95°C for 15 s, 60°C for 60 s, and 95°C for 15s. Expression level was calculated after normalization to the housekeeping gene expression.

### Immunofluorescence Staining

After dewaxing and hydration of paraffin‐embedded sections, the antigen retrieval was conducted for 10 minutes using 0.01 M sodium citrate buffer. The sections were subsequently immersed in 3% H_2_O_2_‐methanol solution for 20 minutes. After washing, the sections were blocked with 1% bovine serum albumin (BSA) for 10 minutes. The sections were then incubated with Alexa Fluor 488-conjugated goat anti-mouse IgG or Alexa Fluor 488-conjugated goat anti-mouse IgM or anti-ACK1 primary antibodies overnight at 4°C. Second day, the sections were washed in PBS with 0.1% Tween 20 for 5 times and then sealed the cover slips with anti-fluorescence quenching agent. The sections were analyzed under a fluorescence microscope (Olympus, Japan).

### Histological Analyses

Paraformaldehyde-fixed lung, liver and kidney tissues were dehydrated in ethanol and paraffin embedded. Tissue sections of 5 μm thickness were stained with 0.1% hematoxylin for 10 min and with 0.5% eosin for 1 min (both at 22 ± 2°C). Histopathological changes were observed using an optical microscope (NikonCorporation, Japan) in randomly selected fields. Histopathologic evaluation lung, liver and kidney tissues histopathologic changes were evaluated using a four-step grading scale based on severity and extent of changes.

### Lentivirus Infections

Lentiviruses expressing ACK1 (ACK1-LV) and lentiviruses expressing ACK1-specific RNAi (RNAi-ACK1-LV) were purchased from GeneChem (Shanghai, China). BMDMs were transfected by using lentivirus according to the manufacturer’s instructions.

### *In Situ* Terminal Deoxynucleotidyl Transferase-Mediated Uridine Triphosphate Nick-End Labeling Assay

TUNEL staining was used to detect DNA fragmentation *in situ* and performed with the *In Situ* Cell Death Detection Kit, TMR red (Roche), according to manufacturer’s instructions. TUNEL positive cells were observed in five randomly-selected fields using a fluorescence microscope; the nucleus is blue and positive apoptotic cells are green.

### Flow Cytometry

Cell suspensions were prepared from cultured *in vitro* or mice model spleens and lymph nodes. The cells were stained with the corresponding antibodies for 30 min at 4°C according to the manufacturer’s instructions. The analysis of the sample was recorded using a FACS Calibur (Becton Dickinson) with forward and side scattered light to establish a gate for intact, live and non-aggregated cells. For each sample, a minimum of 30,000 events were collected in order to achieve a statistically relevant population. All the FACS data were analyzed on FlowJo software.

### Enzyme-Linked Immunosorbent Assay

The concentrations of interleukin-6 (IL-6), interleukin-12 (IL-12), and tumor necrosis factor-α (TNF-α) protein levels secreted by cells were quantified by a sandwich enzyme immunoassay using ELISA kit (Biolegend, USA) following the manufacturer’s instructions. Absorbance at 450 nm was measured using a microplate reader (BioTek microplate reader). All samples were assayed in duplicate.

### Immunoblotting Analysis

In brief, 10 mg protein lysates were run on 8%~12% SDS-PAGE gels and then electrotransferred onto polyvinylidene difluoride (PVDF) membrane in a transfer solution at 100 V for 1 hour. Each membrane was blocked with 3% BSA for 2 hours at room temperature and incubated overnight with primary antibody (1:1000) at 4°C. Next day, after washing, the membranes were incubated with HRP-conjugated secondary antibodies. In the end, the membranes were detected with ECL kit (Thermo, USA) with chemiluminescence.

### Statistical Analysis

All statistical tests have been mentioned in their respective legends, where they can be sided this has been stated. Error bars indicate mean ± standard error of the mean (SEM). Differences between data sets were analyzed by performing ANOVA test or t-test. A p < 0.05 was considered statistically significant (ns, *p* > 0.05, *p < 0.05, **p < 0.01, ***p < 0.001). Survival curves were estimated by using Kaplan–Meier method and the Log-rank test was applied to determine the differences of survival rate. Statistically significant was assumed for *P* < 0.05 (∗), *P* < 0.01 (∗∗), *P* < 0.001 (∗∗∗), and not significant (ns). Plots and charts were constructed and produced by GraphPad Prism 8 (GraphPad Software, Inc.).

## Results

### ACK1 Expression Is Upregulated by TLRs in Macrophages and DCs

**Figure 1:**
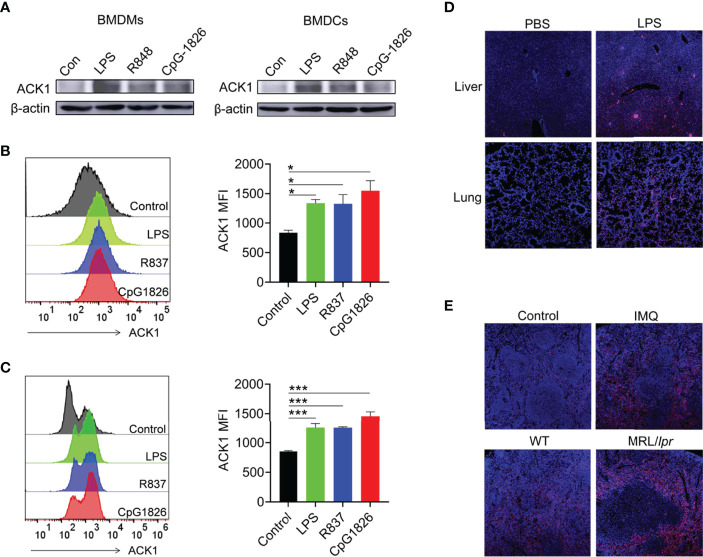
TLRs induced ACK1 expression *in vivo* and *in vitro*. **(A–C)** Murine bone marrow cells-derived macrophages (BMDMs) and dendritic cells (BMDCs) were treated with LPS (100 ng/mL), R848 (1 μg/mL) and CpG-1826 (1 μM) for 24 hours. Western blot was performed to detect expression of ACK1 in BMDMs and BMDCs **(A)**. FACS analysis was performed to assess ACK1 expression in BMDMs **(B)** and BMDCs **(C)** at 24 hours. The data shown represent the means of three independent experiments and the error bars represent the S.E.M. **(D)** C57BL/6 mice were challenged by LPS (10 μg/g body weight) for 12 hours and then the expression of ACK1 in the lung and liver tissue sections was detected by using a confocal microscope. **(E)** Representative image of immunofluorescence of the ACK1 expression in the spleen pathological section from C57BL/6 mice treated-imiquimod for 10 weeks or spontaneous lupus MRL/*lpr* mice (18-week-old). Blue represents DAPI; red represents ACK1. The data are shown as the means ± SEM (n = 6 mice/group) and are representative of three independent experiments. *p < 0.05, ***p < 0.001, as determined by ANOVA tests.

TLRs are pivotal biomolecules in the immune system (32). To investigate whether TLRs can regulate the expression of ACK1 in macrophages and DCs, BMDMs and BMDCs were treated with the TLR4 agonist lipopolysaccharide (LPS), the TLR7 agonist R848, and the TLR9 agonist CpG–1826, and the protein level of ACK1 was detected after 24 hours. As shown in **Figure 1A**, the activation of TLR4/7/9 significantly induced ACK1 expression in macrophages and DCs. The same phenomenon was also observed at the protein level by FACS assay (**Figures 1B, C**). To explore whether TLR4/7/9-induced ACK1 expression is related with p65, the phosphorylation of p65 in BMDMs and BMDCs stimulated with LPS, R848 or CpG-1826 were detected by western blot. As shown in **Figure S1**, compared with the vehicle group, LPS-, R848- and CpG-1826-treated BMDMs and BMDCs showed higher phosphorylation levels of p65, respectively, suggesting that the function of TLR4/7/9 on ACK1 expression maybe dependent on p65.

To verify whether TLRs can regulate the expression of ACK1 *in vivo*, the expression of ACK1 was detected in the liver and lung of LPS-induced endotoxin shock model mice by immunofluorescence and in the spleen of lupus-prone model mice. As expected, ACK1 expression in the liver and lung of endotoxin shock model mice was significantly higher than that in control mice (**Figure 1D**). Moreover, ACK1 expression in the spleens of imiquimod (IMQ)-induced lupus model mice was also higher than that in control mice. The expression of ACK1 was also increased in the spleens of spontaneous lupus MRL/*lpr* mice as compared to that in untreated C57BL/6 mice (**Figure 1E**). These studies demonstrated that the expression of ACK1 could be induced by TLRs *in vivo* and *in vitro*.

### Overexpression of ACK1 Promotes but Knockdown of ACK1 Inhibits the Activation of TLR Pathways in Macrophages

**Figure 2:**
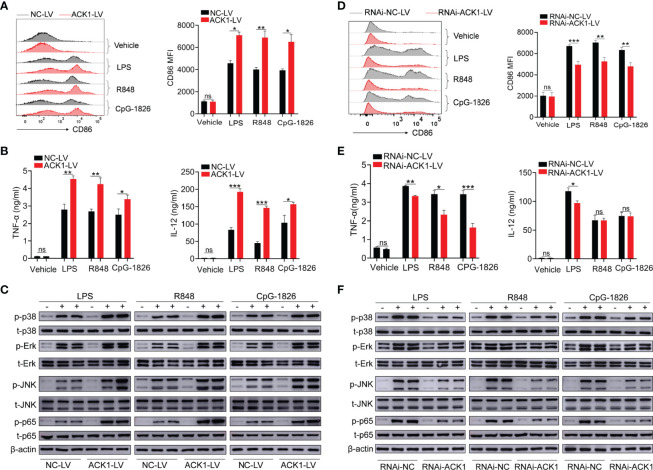
ACK1 augments the activation of BMDMs induced by TLRs. **(A–C)** BMDMs were infected with lentivirus that highly expresses ACK1 (ACK1-LV) or negative control lentivirus (NC-LV) for 3 days followed by stimulation of LPS (100 ng/mL), R848 (1 μg/mL) and CpG-1826 (1 μM). The expression of CD86 **(A)** on BMDMs was detected by flow cytometry at 24 hours. The levels of TNF-α and IL-12 in culture supernatant were detected by ELISA at 24 hours **(B)**. The phosphorylation levels of p38, Erk, JNK and p65 were analyzed by western blot at 1 hour **(C)**. **(D–F)** BMDMs were infected with lentivirus that knockdown ACK1 expression (RNAi-ACK1-LV) or negative control lentivirus (RNAi-NC-LV) for 3 days followed by stimulation of LPS (100 ng/mL), R848 (1 μg/mL) and CpG-1826 (1 μM). The expression of CD86 **(D)** on BMDMs was detected by flow cytometry at 24 hours. The levels of TNF-α and IL-12 in culture supernatant were detected by ELISA at 24 hours **(E)**. The phosphorylation levels of p38, Erk, JNK and p65 were analyzed by western blot at 1 hour **(F)**. The data shown represent the means of three independent experiments and the error bars represent the S.E.M. *p < 0.05, **p < 0.01, and ***p < 0.001, as determined by ANOVA tests; ns denotes *p* > 0.05.

To clarify the role of ACK1 in regulating the activation of TLR pathways, an ACK1-LV lentivirus, a lentivirus with a high expression of ACK1, was used to explore whether ACK1 can regulate the activation of TLR pathways in BMDMs. Based on the analysis of the mRNA and protein level, as shown in **Figures S2A, B**, BMDMs transfected with ACK1-LV showed a higher level of ACK1 expression than those transfected with negative-control lentivirus (NC-LV). Intriguingly, BMDMs transfected with ACK1-LV also showed higher levels of CD86 (**Figure 2A**) induced by LPS, R848, or CpG-1826 than those transfected with NC-LV. Moreover, the levels of IL-12, TNF-α, and IFN-α secreted by BMDMs transfected with ACK1-LV were also higher than those secreted by BMDMs transfected with NC-LV following the treatment with LPS, R848, and CpG-1826 (**Figures 2B** and **S3A**). Mechanistically, the overexpression of ACK1 significantly promoted LPS-, R848-, or CpG-1826-induced phosphorylation of p38, Erk, JNK, and p65 (**Figure 2C**).

To confirm that ACK1 can regulate the activation of TLR pathways, RNAi-ACK1-lentiviruses were used to explore whether knockdown of ACK1 can inhibit the activation of TLRs pathways in BMDMs. As shown in **Figures S2C, D**, BMDMs transfected with RNAi-ACK1-LV showed a lower level of ACK1 expression than those transfected with RNAi-NC-LV. Intriguingly, BMDMs transfected with RNAi-ACK1-LV showed lower levels of CD86 (**Figure 2D**) induced by LPS, R848, or CpG-1826 than those transfected with RNAi-NC-LV. Moreover, the level of TNF-α and IFN-α secreted by BMDMs transfected with RNAi-ACK1-LV was lower than that secreted by BMDMs transfected with RNAi-NC-LV under the effect of LPS, R848, and CpG-1826 (**Figures 2E** and **S3B**). Although there were no difference in the secretion of IL-12 between BMDMs transfected with RNAi-ACK1-LV and RNAi-NC-LV under the effect of R848 and CpG-1826, the level of IL-12 secreted by BMDMs transfected with RNAi-ACK1-LV was lower than that secreted by BMDMs transfected with RNAi-NC-LV under the effect of LPS (**Figure 2E**). Mechanistically, the knockdown of ACK1 significantly inhibited LPS-, R848-, or CpG-1826-induced phosphorylation of p38, Erk, JNK, and p65 (**Figure 2F**). These findings suggest that ACK1 can augment the activation of TLR pathways.

### Inhibition of ACK1 Activity Weakens TLR-Mediated Activation of Macrophages

**Figure 3:**
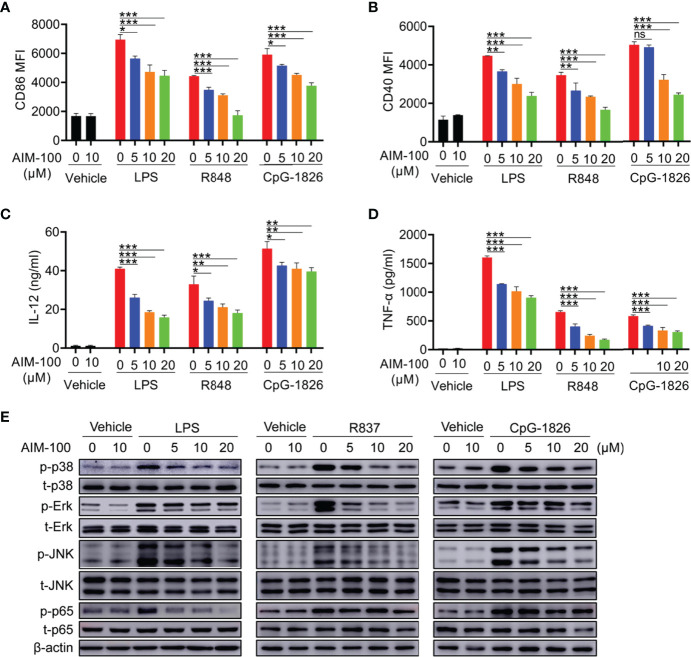
ACK1 inhibitor AIM-100 weakens TLRs-mediated activation of BMDMs. Bone marrow-derived BMDMs from C57BL/6 mice aged 6-8 weeks were pretreated with ACK1 inhibitor AIM-100 (5, 10 and 20 μM) for 2 hours, and were then stimulated with LPS (100 ng/mL), R848 (1 μg/mL) and CpG-1826 (1 μM). FACS analysis of the expressions of CD86 **(A)** and CD40 **(B)** on BMDMs at 24 hours. The levels of IL-12 **(C)** and TNF-α **(D)** secreted by BMDMs were detected by ELISA at 24 hours. Western blot analysis of the phosphorylation levels of p38, Erk, JNK and p65 at 1 hour **(E)**. The data shown represent the means of three independent experiments and the error bars represent the S.E.M. *p < 0.05, **p < 0.01, and ***p < 0.001, as determined by ANOVA tests; ns denotes *p* > 0.05. β-actin was used as loading control.

The ACK1 inhibitor AIM-100 was used to inhibit ACK1 activity to verify the effect of ACK1 on TLR-mediated activation of macrophages. First, we confirmed that AIM-100 at the concentration below 20 µM did not affect the viability of BMDMs (**Figure S4A**). As shown in **Figures 3A, B**, LPS, R848, and CpG-1826 significantly induced the expressions of CD86 and CD40 in vehicle-treated BMDMs, while the expressions of CD86 and CD40 were markedly reduced in AIM-100-treated BMDM as compared to that in vehicle-treated BMDMs following the treatment with LPS, R848, and CpG-1826. Similarly, the expression of IL-12 (**Figure 3C**) and TNF-α (**Figure 3D**) induced by LPS, R848, or CpG-1826 in BMDMs was markedly inhibited by AIM-100.

Next, the effects of ACK1 on the TLR4/TLR7/TLR9-induced activation of the MAPK and NF-kB pathways were detected in BMDMs. As shown in **Figure 3E**, western blot analyses confirmed that the inhibition of ACK1 activity significantly inhibited LPS-, R848-, or CpG-1826-induced phosphorylation of p38, Erk, JNK, and p65 in a dose-dependent manner. In summary, these findings indicate that the inhibition of ACK1 activity can reduce TLR-mediated activation of macrophages.

### Inhibition of ACK1 Activity Weakens TLR-Mediated Activation of DCs

**Figure 4:**
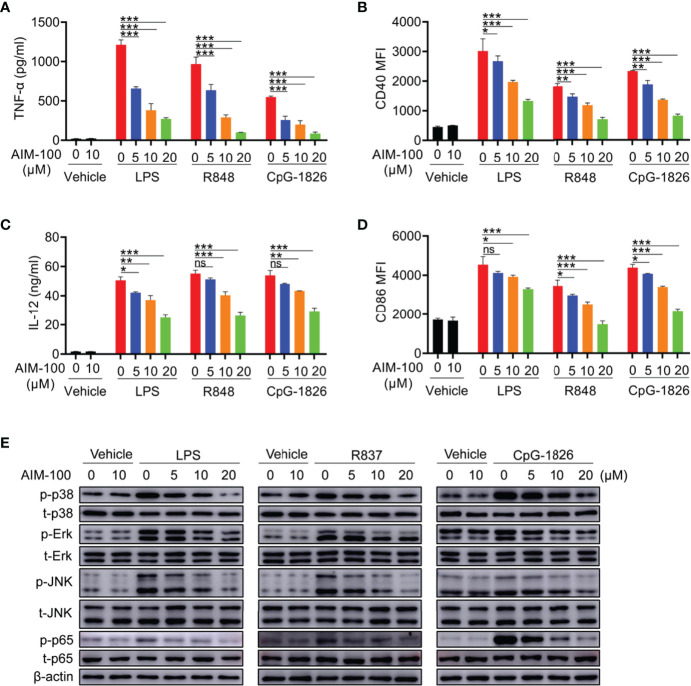
ACK1 inhibitor weakens TLR-mediated activation of DCs. Bone marrow-derived BMDCs from C57BL/6 mice aged 6-8 weeks were pretreated with ACK1 inhibitor AIM-100 (5, 10 and 20 μM) for 2 hours, and were then stimulated with LPS (100 ng/mL), R848 (1 μg/mL) and CpG-1826 (1 μM). FACS analysis of the expressions of CD86 **(A)** and CD40 **(B)** on BMDCs at 24 hours. The levels of IL-12 **(C)** and TNF-α **(D)** secreted by BMDCs were detected by ELISA at 24 hours. Western blot analysis of the phosphorylation levels of p38, Erk, JNK and p65 at 1 hour **(E)**. The data shown represent the means of three independent experiments and the error bars represent the S.E.M. *p<0.05, **p<0.01, and ***p<0.001, as were determined by ANOVA tests; ns denotes *p*>0.05. β-actin was used as loading control.

The effect of AIM-100 on TLR-mediated activation of DCs was also investigated. First, we confirmed that AIM-100 at the concentration below 20 µM did not affect the viability of DCs (**Figure S4B**). As shown in **Figures 4A, B**, LPS, R848, and CpG-1826 significantly induced the expression of CD86 and CD40 in vehicle-treated BMDCs, while the expression of CD86 and CD40 was markedly reduced in AIM-100-treated DCs as compared to that in vehicle-treated DCs following the treatment with LPS, R848, and CpG-1826. Similarly, the expression of IL-12 (**Figure 4C**), TNF-α (**Figure 4D**) and IFN-α (**Figure S3C**) induced by LPS, R848, or CpG-1826 in DCs was markedly inhibited by AIM-100. As expected, western blot analyses confirmed that the inhibition of ACK1 activity significantly inhibited LPS-, R848-, or CpG-1826-induced phosphorylation of p38, Erk, JNK, and p65 in a dose-dependent manner in DCs (**Figure 4E**). In summary, these findings indicate that the inhibition of ACK1 activity can reduce TLR-mediated activation of DCs.

### Pharmaceutical Inhibition of ACK1 Relieves LPS-Induced Endotoxin Shock

**Figure 5:**
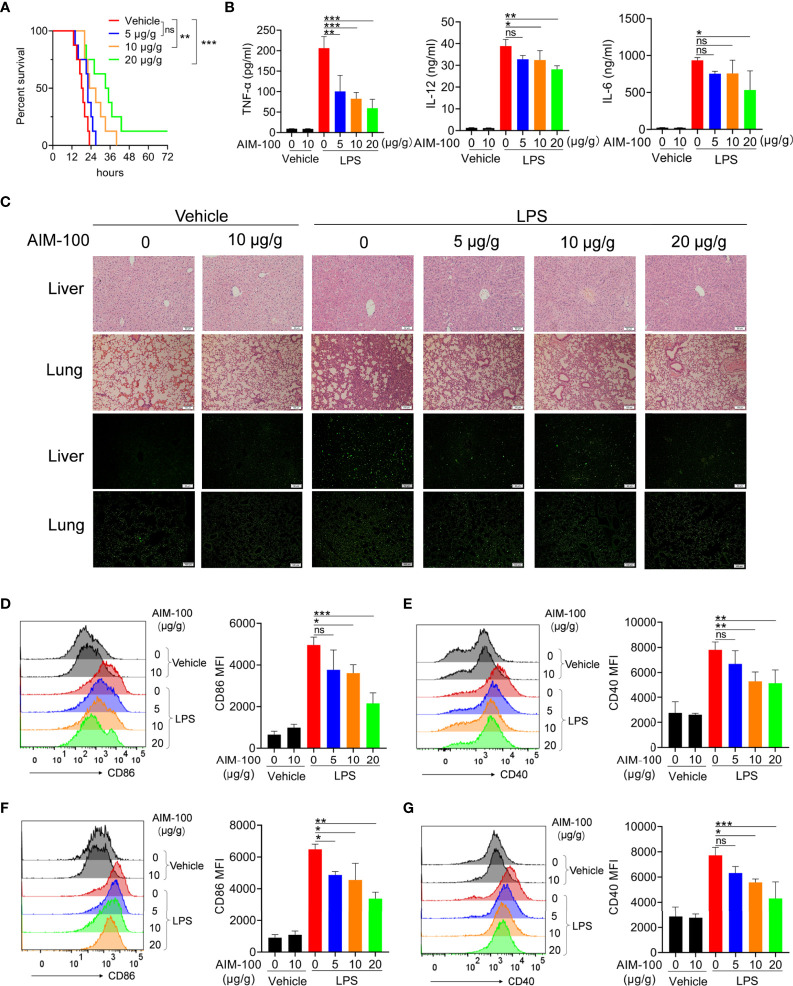
Pharmacological inhibition of ACK1 ameliorates mice with endotoxic shock. **(A)** C57BL/6 mice were injected AIM-100 (5, 10, 20 μg/g body weight) or vehicle for 2 hours followed by LPS challenge (37.5 μg/g of body weight), and the mortality of mice were observed (n = 8 mice/group). Survival curves were estimated by using Kaplan–Meier method and the Log-rank test was applied to determine the differences of survival rate. Statistically significant was assumed for *P* < 0.05 (∗), *P* < 0.01 (∗∗), *P* < 0.001 (∗∗∗), and ns, not significant. **(B–G)** C57BL/6 mice were injected AIM-100 (5, 10, 20 μg/g body weight) or vehicle for 2 hours followed by LPS challenge (10 μg/g of body weight). ELISA measures the levels of TNF-α, IL-6 and IL-12 in serum at 3 hours **(B)**. The lungs and livers of the endotoxic shock model mice were soaked in 4% paraformaldehyde and then paraffin embedded were stained with H&E at 12 hours **(C)**. TUNEL analysis of apoptosis of lung and liver cells at 12 hours **(C)**. The expression levels of CD40 and CD86 on splenic F4/80^+^ macrophages **(D, E)** and CD11c^+^ DCs **(F, G)** of endotoxin shock mice were detected by flow cytometry at 12 hours. The data are shown as the means ± SEM (n=6 mice/group) and are representative of three independent experiments. *p < 0.05, **p < 0.01, ***p < 0.001, as determined by ANOVA tests; ns denotes *p* > 0.05.

Hyper-activation of the TLR4 pathway can lead to endotoxic shock by promoting the abundant secretion of proinflammatory cytokines (33, 34). Given that ACK1 can augment the activation of the TLR4 pathway, we elucidated whether the inhibition of ACK1 activity relieves LPS-induced endotoxin shock. As shown in **Figure 5A**, the mortality of AIM-100-treated mice challenged by LPS was significantly lower than that of vehicle-treated mice challenged by LPS. Moreover, the higher the dose of AIM-100, the better was the survival of mice within a certain dose range. The levels of TNF-α, IL-6 and IL-12 in the serum of AIM-100-treated endotoxic shock mice were also significantly reduced as compared to that of vehicle-treated endotoxic shock mice (**Figure 5B**). Hematoxylin and eosin (H&E) staining showed that AIM-100 significantly alleviated LPS-induced liver and lung injury. The number of apoptotic cells in lung and liver tissues of AIM-100-treated endotoxic shock mice was also significantly lower than that of vehicle-treated endotoxic shock mice (**Figures 5C** and **S5**).

Considering that AIM-100 reduced the mortality and inflammatory levels in endotoxin shock mice, we assumed that the alleviation of endotoxin shock mice might be due to the inhibitory function of AIM-100 on the activation of macrophages and DCs. As expected, the expression levels of CD86 and CD40 in the splenic macrophages (**Figures 5D, E**) and DCs (**Figures 5F, G**) from the AIM-100-treated endotoxic shock mice were significantly lower than those from the vehicle-treated endotoxic shock mice. What’s more, AIM-100-treated endotoxic shock mice showed significantly lower levels of CD86 and CD40 in the macrophages (**Figures S6A, B**) and DCs (**Figures S6C, D**) of lymph nodes compared with those from the vehicle-treated endotoxic shock mice. Taken together, pharmaceutical inhibition of ACK1 relieves LPS-induced endotoxin shock.

### Pharmaceutical Inhibition of ACK1 Relieves TLR7 Ligand IMQ-Induced Lupus

**Figure 6:**
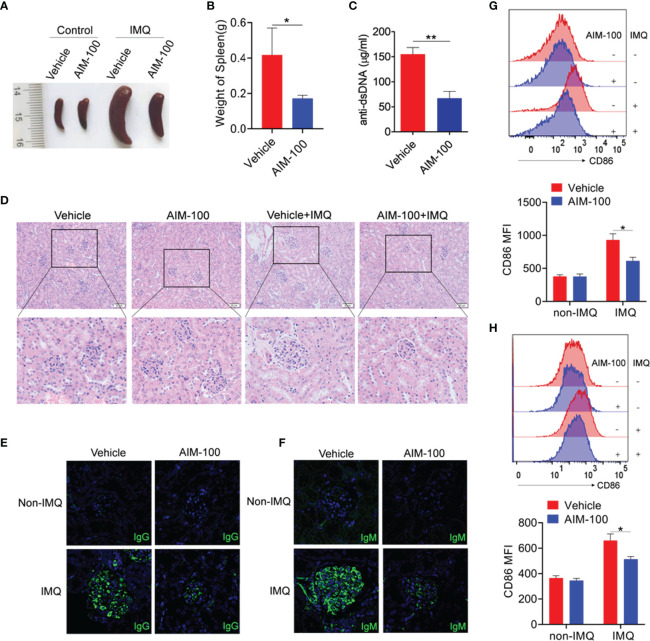
The ACK1 inhibitor AIM-100 alleviates the conditions of IMQ-induced lupus-prone mice. **(A)** The representative spleen images in all group of mice. **(B)** The weight of spleens in IMQ mice treated with vehicle or AIM-100. **(C)** ELISA was performed to analysis of anti-dsDNA antibodies in serum. **(D)** H&E staining of the kidney sections from all groups of mice. **(E, F)** The IgG **(E)** and IgM **(F)** deposit in the kidney sections was detected by immunofluorescence. The scar strip represents 50 μm. **(G, H)** FACS analysis of the expression of CD86 on splenic macrophages **(G)** and DCs **(H)**. Results represented as mean ± SEM (n=6 mice/group). *p < 0.05, **p < 0.01, as determined by ANOVA or Student’s *t* test.

We next investigated whether the inhibition of ACK1 activity can relieve the lesions of IMQ-induced lupus-prone mice (IMQ-mice). After systemic treatment with IMQ, the spleens of IMQ-mice were observed to be significantly larger than those of the control mice (**Figures 6A, B**). Intriguingly, the spleens of AIM-100-treated IMQ-mice were much smaller than those of vehicle-treated IMQ-mice (**Figures 6A, B**). The serum level of anti-dsDNA antibody was also reduced in AIM-100-treated IMQ-mice as compared to that in vehicle-treated IMQ-mice (**Figure 6C**). Compared with vehicle-treated IMQ-mice, AIM-100-treated IMQ-mice showed significant inhibition in the infiltration of lymphoid cells and a diffused expansion of the mesangial matrix in the kidneys (**Figure 6D**). In addition, AIM-100-treated IMQ-mice showed a relatively lesser amount of glomerular deposition of IgG (**Figure 6E**) and IgM (**Figure 6F**) than vehicle-treated IMQ-mice.

Importantly, the activation levels of macrophages and DCs were investigated in AIM-100-treated IMQ-mice and vehicle-treated IMQ-mice. As expected, AIM-100-treated IMQ-mice showed reduced expression levels of the activation markers CD86 on splenic macrophages (**Figure 6G**) and DCs (**Figure 6H**) as compared to vehicle-treated IMQ-mice. Considering that AIM-100 could reduce the serum level of dsDNA in IMQ-mice, the role of AIM-100 in the formation of abnormal germinal center (GC) was also detected. As shown in **Figure S7**, AIM-100-treated IMQ-mice showed a lower percentage of GC B cells (CD95^+^GL7^+^) in B220^+^ B cells and follicular helper T (Tfh) cells (CXCR5^+^PD-1^+^) in CD4^+^ T cells in the spleen and mesenteric lymph nodes (mLNs) as compared to vehicle-treated IMQ-mice. All these results indicate that AIM-100 can effectively exert its effect on mice induced by the TLR7 agonist IMQ.

### Pharmaceutical Inhibition of ACK1 Relieves the Conditions of Lupus-Prone MRL/lpr Mice

**Figure 7:**
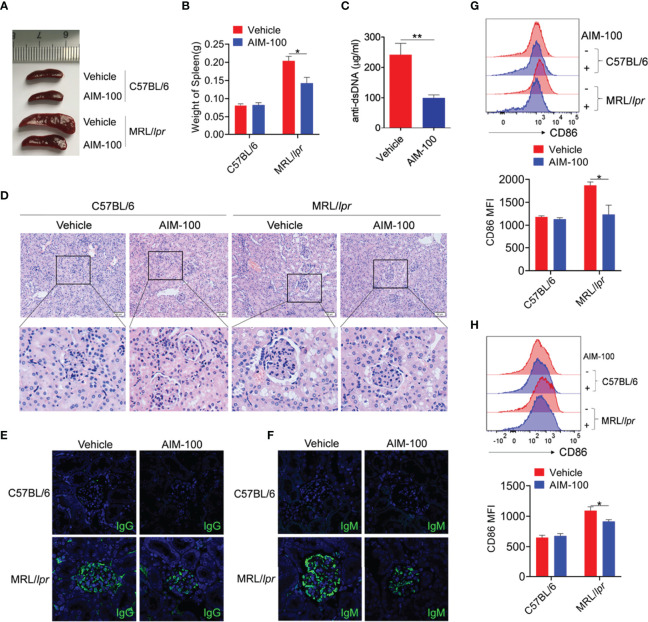
The ACK1 inhibitor AIM-100 alleviates the conditions of MRL/*lpr* lupus-prone mice. **(A)** The representative spleen images in all group of mice. **(B)** The weight of spleens in all group of mice. **(C)** ELISA was performed to analysis of anti-dsDNA antibodies in serum. **(D)** H&E staining of the kidney sections from all groups of mice. **(E, F)** The IgG **(E)** and IgM **(F)** deposit in the kidney sections was detected by immunofluorescence. The scar strip represents 50 μm. **(G, H)** FACS analysis of the expression of CD86 on splenic macrophages **(G)** and DCs **(H)**. Results represented as mean ± SEM (n=5 mice/group). *p < 0.05, **p < 0.01, as determined by ANOVA or Student’s *t* test.

Next, we explored the effect of AIM-100 on the disease onset of MRL/*lpr* lupus-prone mice, a classical mouse model of systemic lupus erythematosus (SLE). Similar to previous findings, the spleens of AIM-100-treated MRL/*lpr* mice were much smaller and lighter than those of vehicle-treated MRL/*lpr* mice (**Figures 7A, B**). The serum level of anti-dsDNA antibody was reduced in AIM-100-treated MRL/*lpr* mice as compared to that in vehicle-treated MRL/*lpr* mice (**Figure 7C**). Furthermore, compared to vehicle-treated MRL/*lpr* mice, AIM-100-treated MRL/*lpr* mice showed significant inhibition in the infiltration of lymphoid cells and a diffused expansion of the mesangial matrix in the kidneys (**Figure 7D**). Moreover, AIM-100-treated MRL/*lpr* mice showed a relatively lesser amount of glomerular deposition of IgG (**Figure 7E**) and IgM (**Figure 7F**) than vehicle-treated MRL/*lpr* mice.

**Figure 8:**
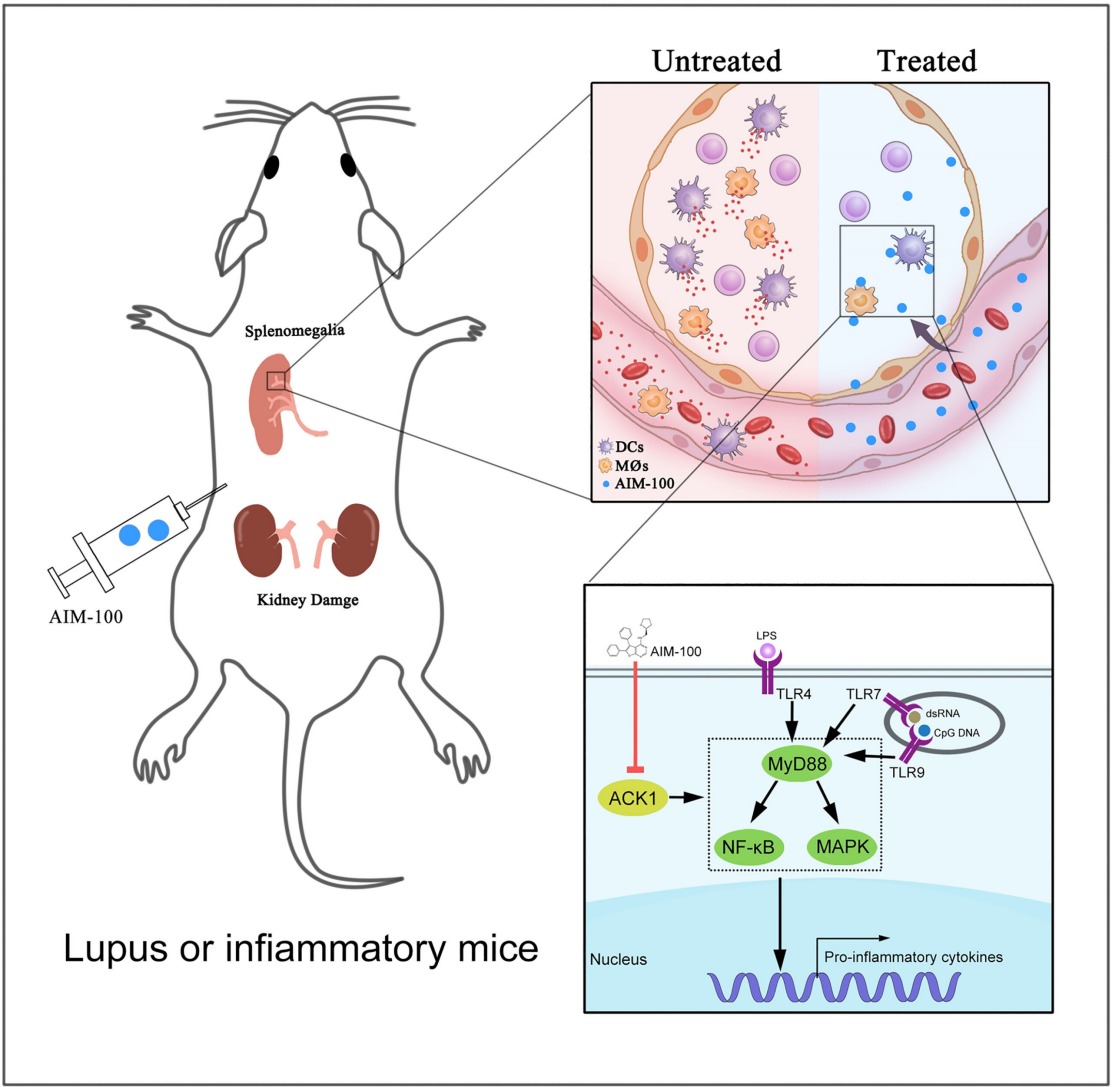
A mechanistic model of ACK1 participates in the pathogenesis of inflammation and autoimmunity by promoting the activation of TLR signaling pathways. This schematic diagram indicted that ACK1 promotes TLR4/TLR7/TLR9-induced activation of macrophages and DCs, therefore contributes to the pathogenesis of TLR-mediated inflammation and autoimmunity; AIM-100, an inhibitor of ACK1, can significantly inhibit TLR4/TLR7/TLR9-induced activation of macrophages and DCs, and alleviates the TLRs-mediated inflammation and autoimmunity.

The activation levels of macrophages and DCs were investigated in AIM-100-treated MRL/*lpr* mice and vehicle-treated MRL/*lpr* mice. As expected, AIM-100-treated MRL/*lpr* mice showed reduced expression levels of the activation markers CD86 on splenic macrophages (**Figure 7G****)** and DCs (**Figure 7H**) as compared to vehicle-treated MRL/*lpr* mice. As T cells play a critical role in the pathogenesis of MRL/*lpr* mice, we also investigated the activation level of T cells in AIM-100-treated MRL/*lpr* mice and vehicle-treated MRL/*lpr* mice. As shown in **Figure S7**, AIM-100-treated MRL/*lpr* mice showed a significantly lower level of CD69 expression on splenic CD4^+^ T cells compared with vehicle-treated MRL/*lpr* mice. More importantly, AIM-100-treated MRL/*lpr* mice showed a lower percentage of Tfh cells in splenic CD4^+^ T cells as compared to vehicle-treated MRL/*lpr* mice (**Figure S8**). All these results indicate that AIM-100 can effectively relieve the conditions of lupus-prone MRL/*lpr* mice. Taken together, these results confirmed our hypothesis that ACK1 promotes TLR4/TLR7/TLR9-induced activation of macrophages and DCs, therefore contributes to the pathogenesis of TLR-mediated inflammation and autoimmunity. AIM-100, an inhibitor of ACK1, can significantly inhibit TLR4/TLR7/TLR9-induced activation of macrophages and DCs and alleviates the TLRs-mediated inflammation and autoimmunity (**Figure 8**). These findings will help us deepen understanding of the pathogenesis of inflammation and autoimmunity, and provide potential targets for the control of TLR-related diseases.

## Discussion

ACK1, a tyrosine kinase, participates in the regulation of many signaling pathways and performs corresponding physiological functions; thus, influencing several important cellular processes, cell proliferation, movement, and cell division cycle (35–38). To date, only one study has reported the correlation between ACK1 and immunity, that is, the immune cell infiltration level is related to the ACK1 gene copy number in lung cancer (39). Thus, its regulatory effect on the TLR pathway remains poorly understood. Our present study is the first to report that ACK1 is involved in regulating the activation of the TLR signaling pathways and mediating inflammation and autoimmunity, and that inhibition of ACK1 activity can significantly attenuate the pathogenesis of inflammatory and autoimmune diseases.

It has been reported that ACK1 is highly expressed in patients with various tumors and colitis (40), but the regulatory mechanism of ACK1 expression remains unclear. The expression of ACK1 in patients with inflammatory and autoimmune diseases has, however, not yet been reported. We found that TLR4, TLR7, and TLR9 pathways could induce the expression of ACK1 in macrophages and DCs to varying degrees at the cellular level. We also found that the expression of ACK1 was abnormally increased in mice treated with the TLR4 agonist LPS and the TLR7 agonist IMQ; thus, confirming that TLRs can contribute to the expression of ACK1.

As a tyrosine kinase, ACK1 plays an important role in the regulation of many signaling pathways such as cell proliferation, movement, and cell division cycle. However, its role in the regulation of the TLR pathway remains unclear. We found that ACK1 is involved in regulating the activation of the TLR signaling pathway and in mediating inflammation and autoimmunity, and we elucidated the molecular mechanism by which ACK1 regulates the activation of the TLR signaling pathway. At the cellular level, we used ACK1 high-expressing lentivirus, interfering lentivirus, and ACK1 inhibitors to study the effect of ACK1 on the activation of the TLR pathway. ACK1 was found to augment the activation of TLR pathways. In *in vivo* studies, we investigated the effect of the ACK1 inhibitor AIM-100 on TLR4 agonist LPS-mediated endotoxic shock model mice and lupus-prone model mice. We believe that it will be more meaningful for researchers to use ACK1 knockout mice to further study the regulatory function of ACK1 on TLR4 agonist LPS-mediated endotoxic shock model mice and lupus-prone model mice.

TLRs can induce a series of inflammatory responses by activating the MAPK and NF-κB pathways. In the present study, we discovered that ACK1 could significantly promote TLRs-induced activation of the MAPK and NF-κB pathways. However, it should be noted that we did not investigate in depth how ACK1 regulates TLR-induced activation of the MAPK and NF-κB pathways. Therefore, further studies are needed to explore the specific mechanism by which ACK1 regulates TLR activation.

It is noteworthy that ACK1 inhibitors attenuated acute inflammation and lupus in model mice, which suggests that ACK1 may be a promising target for the treatment of inflammation and lupus. Moreover, because ACK1 inhibitors could significantly inhibit the activation of the TLR signaling pathway, we speculate that ACK1 inhibitors may have therapeutic effects on other diseases related to TLR pathways. This hypothesis needs further studies for confirmation.

## Data Availability Statement

The raw data supporting the conclusions of this article will be made available by the authors, without undue reservation.

## Ethics Statement

Guiding Principles for the Care and Use of Laboratory Animals reviewed and approved by the Jining Medical University Animal Care Committee.

## Author Contributions

LJ, XZ, YY, and GD performed the experiments. LJ, XZ, DL, and GD analyzed the data and generated figures. LJ, HX, and GD wrote the manuscript. All authors contributed to the article and approved the submitted version.

## Funding

This work was supported by the National Natural Science Foundation of China (NO. 82071824 and 81901655), Project of Shandong Province Higher Educational Youth Innovation Science and Technology Program (2021KJ074).

## Conflict of Interest

The authors declare that the research was conducted in the absence of any commercial or financial relationships that could be construed as a potential conflict of interest.

## Publisher’s Note

All claims expressed in this article are solely those of the authors and do not necessarily represent those of their affiliated organizations, or those of the publisher, the editors and the reviewers. Any product that may be evaluated in this article, or claim that may be made by its manufacturer, is not guaranteed or endorsed by the publisher.
