# Supplementary material for: ACK1 Contributes to the Pathogenesis of Inflammation and Autoimmunity by Promoting the Activation of TLR Signaling Pathways
Source: Front Immunol. 2022 May 20;13:864995. doi: 10.3389/fimmu.2022.864995 (PMC9164107; doi:10.3389/fimmu.2022.864995)
Supplement: Supplementary file 1 [file DataSheet_1.docx]

**Supplemental Data**

**
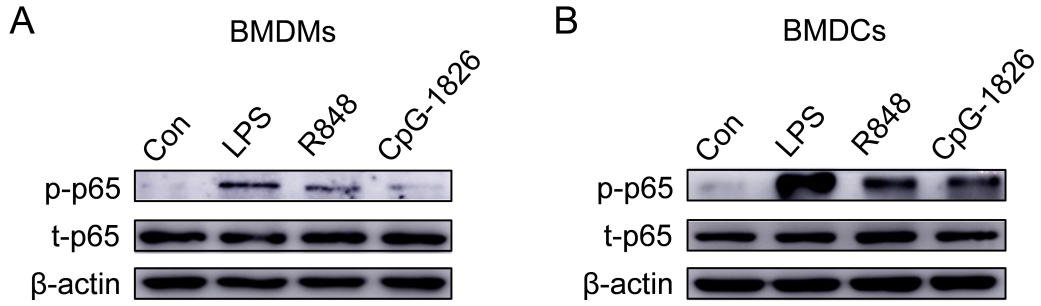
**

FIGURE S1 TLRs induced phosphorylation of p65 BMDMs and BMDCs. Murine BMDMs and BMDCs were treated with LPS (100 ng/mL), R848 (1 μg/mL) and CpG-1826 (1 μM) for 1 hour. Western blot was performed to detect the phosphorylation of p65 in BMDMs **(A)** and BMDCs **(B)**. The data shown represent the means of three independent experiments and β-actin was used as loading control.


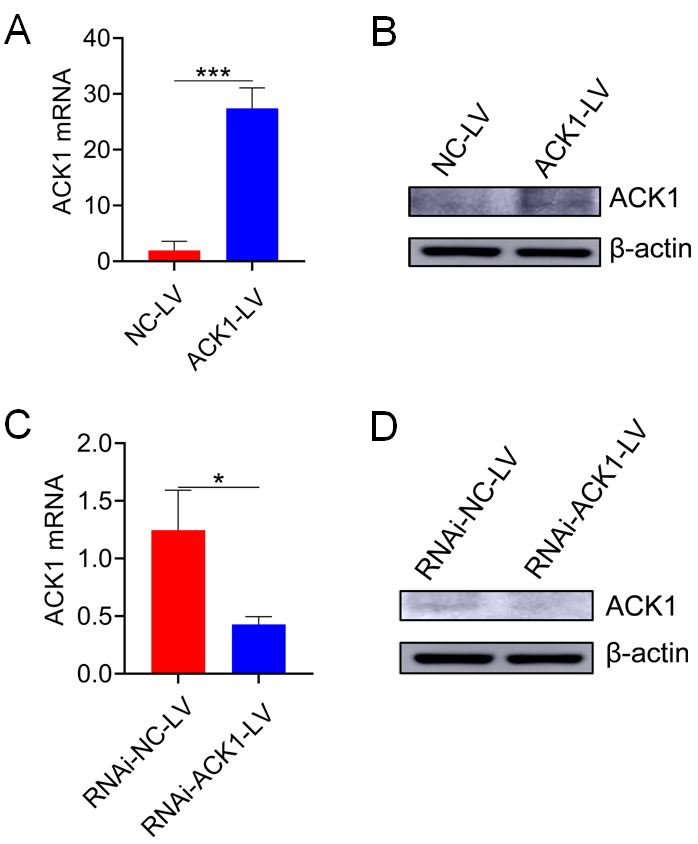


FIGURE S2 The expression of ACK1 in BMDMs infected with ACK1-LV and RNAi-ACK1-LV. **(A, B)** qPCR and western blot analysis of the expression of ACK1 in BMDMs infected with ACK1-LV or NC-LV for 3 days. **(C, D)** qPCR and western blot analysis of the expression of ACK1 in BMDMs infected with RNAi-ACK1-LV or RNAi-NC-LV for 3 days. The data shown represent the means of three independent experiments and the error bars represent the S.E.M.. **p*<0.05, ****p*<0.001, as determined by *t*-test.

**
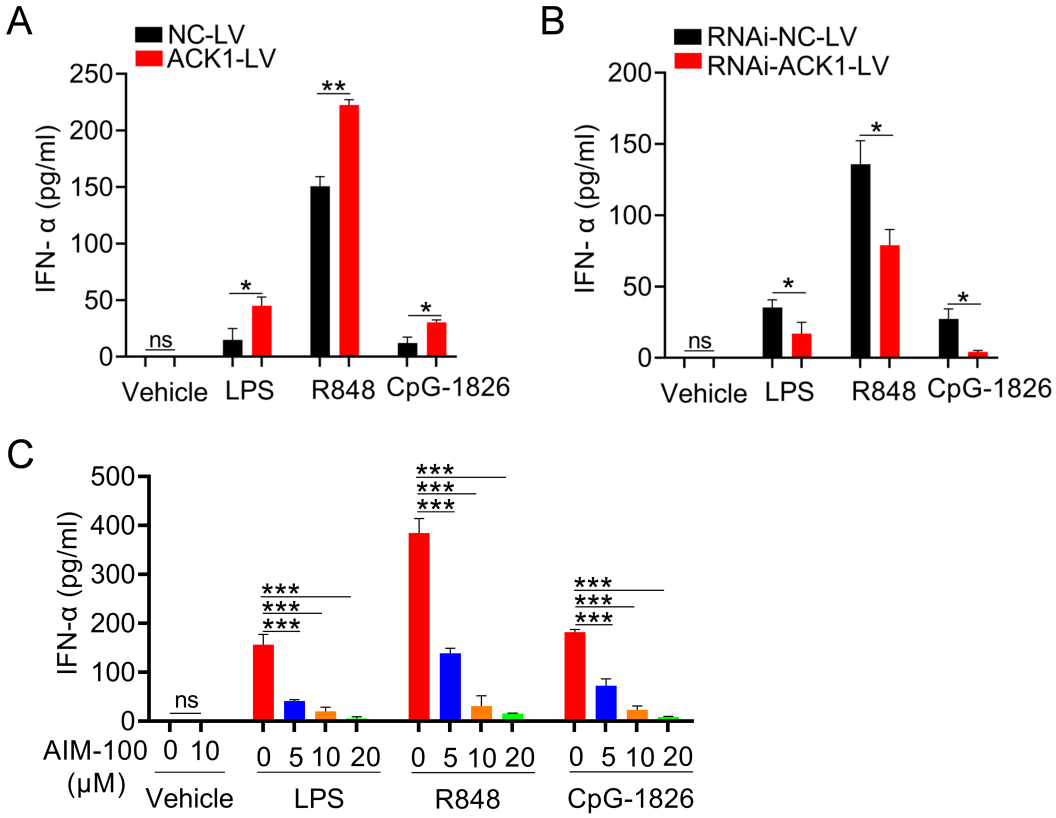
**

FIGURE S3 The effect of ACK1 on the TLR4/7/9-induced secretion of IFN-α. **(A)** BMDMs were infected with ACK1-LV or NC-LV for 3 days followed by stimulation of LPS (100 ng/mL), R848 (1 μg/mL) and CpG-1826 (1 μM). The level of IFN-α in culture supernatant was detected by ELISA at 24 hours. **(B)** BMDMs were infected with RNAi-ACK1-LV or RNAi-NC-LV for 3 days followed by stimulation of LPS (100 ng/mL), R848 (1 μg/mL) and CpG-1826 (1 μM). The level of IFN-α in culture supernatant was detected by ELISA at 24 hours. **(C)** BMDCs were pretreated with ACK1 inhibitor AIM-100 (5, 10 and 20 μM) for 2 hours, and were then stimulated with LPS (100 ng/mL), R848 (1 μg/mL) and CpG-1826 (1 μM). The level of IFN-α in culture supernatant was detected by ELISA at 24 hours. The data shown represent the means of three independent experiments and the error bars represent the S.E.M.. **p*<0.05, ***p*<0.01, ****p*<0.001, as determined by *t*-test; ns denotes *p*>0.05.

**
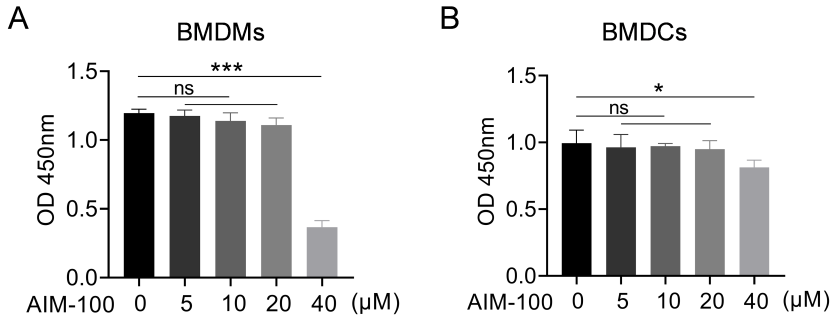
**

FIGURE S4 The effect of AIM-100 on the viabilities of BMDMs and BMDCs isolated from tibias and femurs of C57BL/6 mice. BMDMs and BMDCs were treated with AIM-100 (0, 5, 10, 20, and 40 μM) for 24 hours. Cell viability was monitored with CCK8 kit following the instructions. The data shown represent the means of three independent experiments and the error bars represent the S.E.M.. **p*<0.05, ****p*<0.001, as determined by ANOVA tests; ns denotes *p*>0.05.


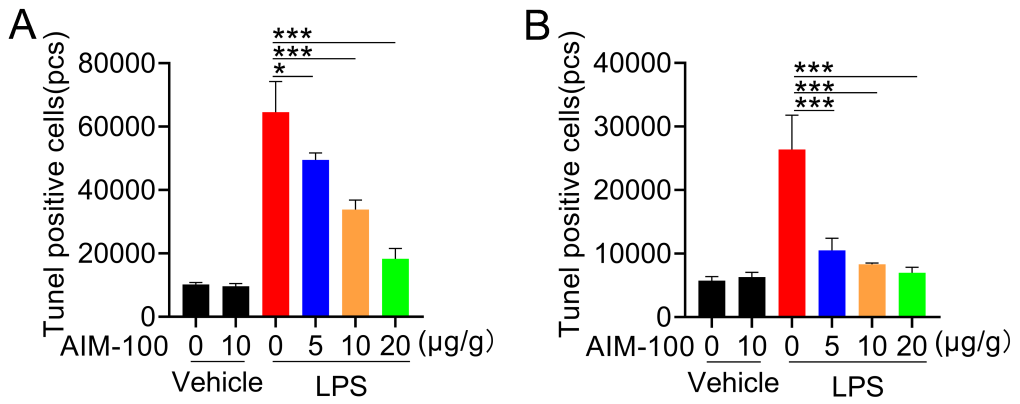


FIGURE S5 Pharmacological inhibition of ACK1 reduces the apoptosis of lung and liver cells in mice with endotoxic shock. C57BL/6 mice were injected AIM-100 (5, 10, 20 μg/g body weight) or vehicle for 2 hours followed by LPS challenge (10 μg/g of body weight). TUNEL was used to detect the apoptosis of liver and lung cells at 12 hours. The TUNEL pictures of the same tissue were placed in the same file, and the pictures were analyzed by Image J and the number of positive cells in the TUNEL sections of the livers (A) and lungs (B) was analyzed by Prism 8. The data are shown as the means ± SEM (n=6 mice/group) and are representative of three independent experiments. **p*<0.05, ****p*<0.001, as determined by ANOVA tests; ns denotes *p*>0.05.


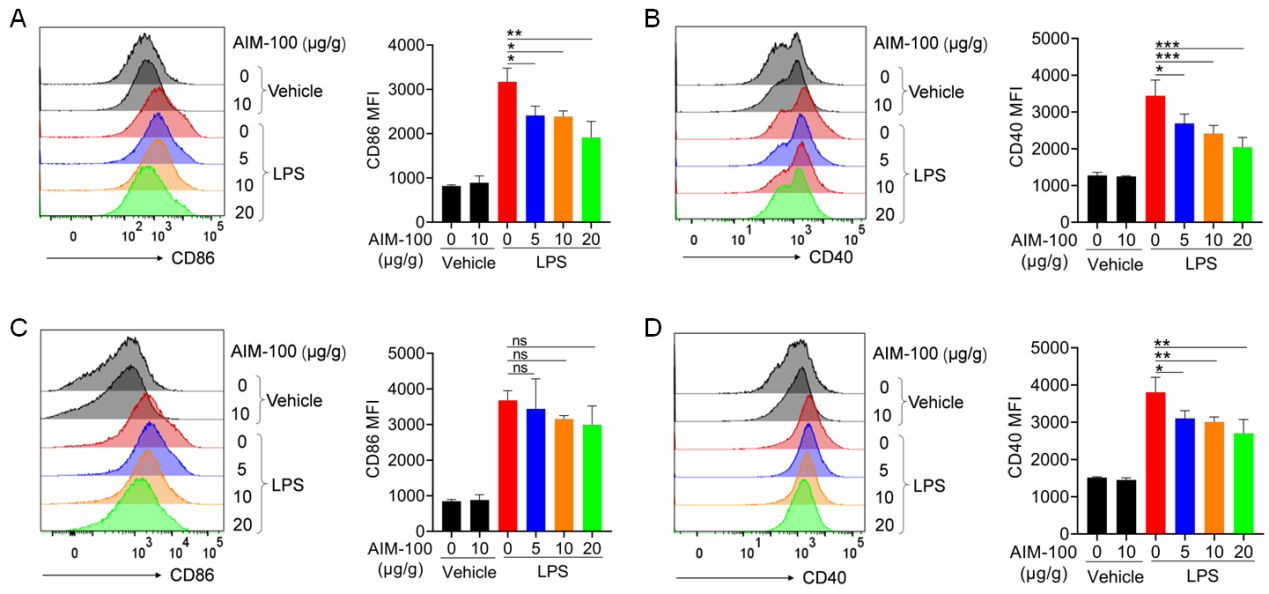


FIGURE S6 Pharmaceutical inhibition of ACK1 relieves the activation of macrophages and DCs in mesenteric lymph nodes of endotoxin shock mice. C57BL/6 mice were injected AIM-100 (5, 10, 20 μg/g body weight) or vehicle for 2 hours followed by LPS challenge (10 μg/g of body weight). The expression levels of CD40 and CD86 on splenic F4/80^+^ macrophages **(A, B)** and CD11c^+^ DCs **(C, D)** of endotoxin shock mice were detected by flow cytometry at 12 hours. The data are shown as the means ± SEM (n=6 mice/group) and are representative of three independent experiments. **p*<0.05, ***p*<0.01, ****p*<0.001, as determined by ANOVA tests; ns denotes *p*>0.05.


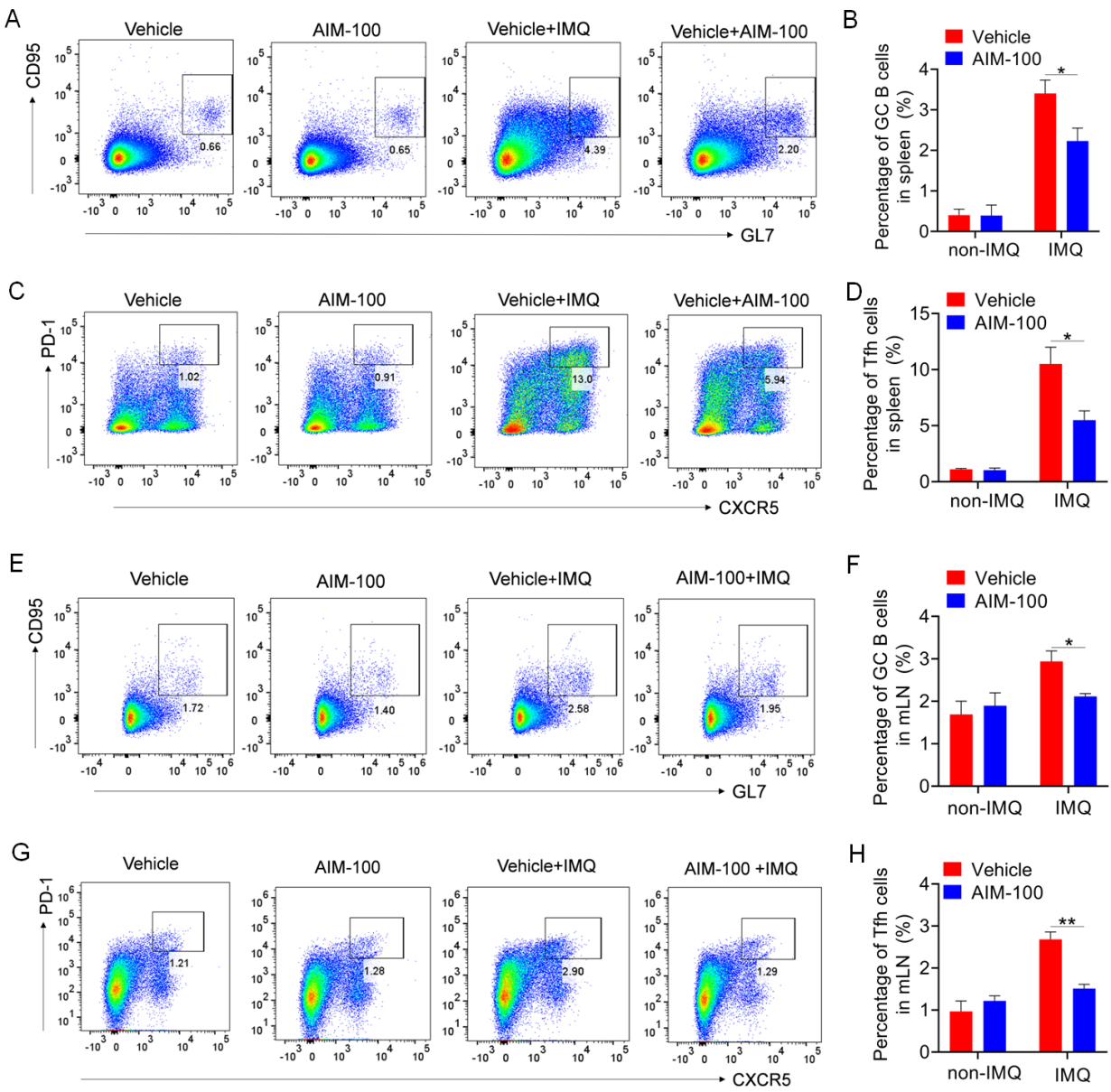


FIGURE S7 Pharmaceutical inhibition of ACK1 reverses germinal center formation in IMQ-treated mice. (**A-D**) FACS analysis of the percentages of CD95^+^GL7^+^ GC B cells in B220^+^ B cells (**A, B**) and CXCR5^+^PD-1^+^ Tfh cells in CD4^+^ T cells (**C, D**) from the spleens of all groups of mice. (**E-H**) FACS analysis of the percentages of CD95^+^GL7^+^ GC B cells in B220^+^ B cells (**E, F**) and CXCR5^+^PD-1^+^ Tfh cells in CD4^+^ T cells (**G, H**) from the mesenteric lymph nodes of all groups of mice. The data are shown as the means ± SEM (n=6 mice/group) and are representative of three independent experiments. **p*<0.05, ***p*<0.01, as determined by ANOVA tests.


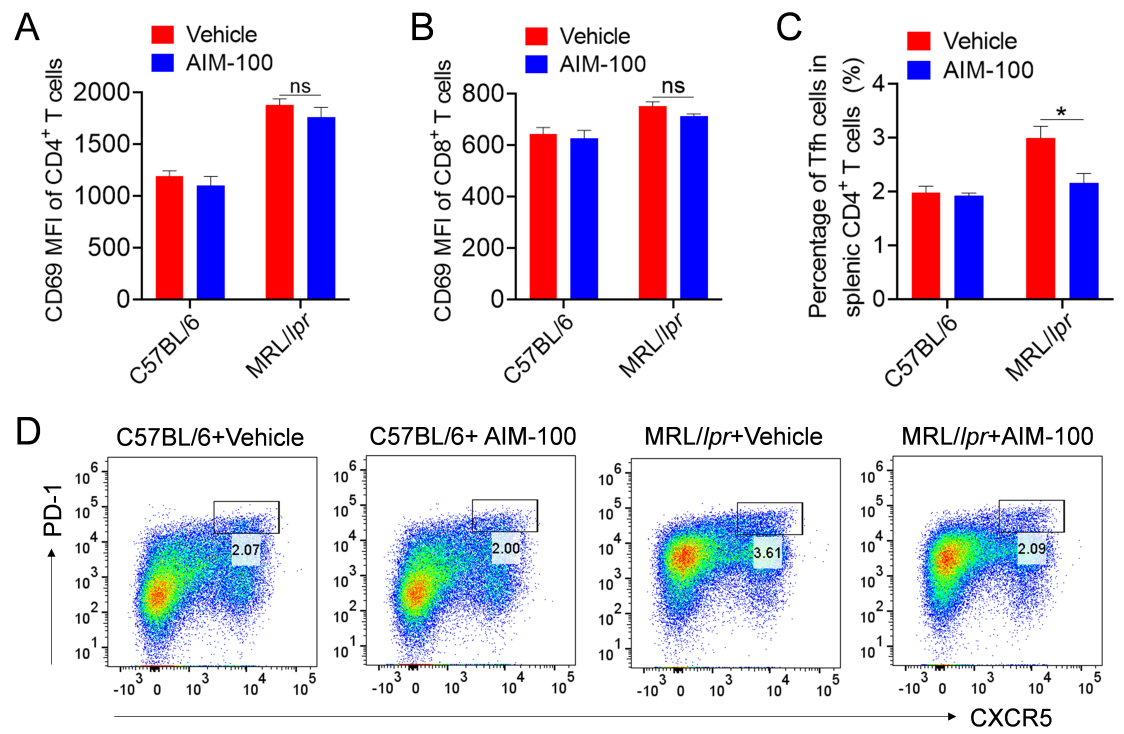


FIGURE S8 The effect of AIM-100 on the activation of T cells and the differentiation of Tfh in MRL/*lpr* lupus-prone mice. **(A)** FACS analysis of the expression of CD69 on splenic CD4^+^ T cells**. (B)** FACS analysis of the expression of CD69 on splenic CD8^+^ T cells**. (C, D)** FACS analysis of the percentage of Tfh in splenic CD4^+^ T cells**.** Results represented as mean ± SEM (n=5 mice/group). **p* < 0.05, as determined by ANOVA; ns denotes *p*>0.05.
